# Impact of Language Experience on Attention to Faces in Infancy: Evidence From Unimodal and Bimodal Bilingual Infants

**DOI:** 10.3389/fpsyg.2018.01943

**Published:** 2018-10-16

**Authors:** Evelyne Mercure, Isabel Quiroz, Laura Goldberg, Harriet Bowden-Howl, Kimberley Coulson, Teodora Gliga, Roberto Filippi, Peter Bright, Mark H. Johnson, Mairéad MacSweeney

**Affiliations:** ^1^Institute of Cognitive Neuroscience, University College London, London, United Kingdom; ^2^Centre for Brain and Cognitive Development, Birkbeck, University of London, London, United Kingdom; ^3^School of Psychology, University of Plymouth, Plymouth, United Kingdom; ^4^Department of Psychology and Sports Sciences, University of Hertfordshire, Hatfield, United Kingdom; ^5^Institute of Education, University College London, London, United Kingdom; ^6^Department of Psychology, Anglia Ruskin University, Cambridge, United Kingdom; ^7^Department of Psychology, University of Cambridge, Cambridge, United Kingdom

**Keywords:** infants, bilingualism, Deaf, sign language, face processing, eye-tracking, bimodal bilingualism, visual attention

## Abstract

Faces capture and maintain infants’ attention more than other visual stimuli. The present study addresses the impact of early language experience on attention to faces in infancy. It was hypothesized that infants learning two spoken languages (unimodal bilinguals) and hearing infants of Deaf mothers learning British Sign Language and spoken English (bimodal bilinguals) would show enhanced attention to faces compared to monolinguals. The comparison between unimodal and bimodal bilinguals allowed differentiation of the effects of learning two languages, from the effects of increased visual communication in hearing infants of Deaf mothers. Data are presented for two independent samples of infants: Sample 1 included 49 infants between 7 and 10 months (26 monolinguals and 23 unimodal bilinguals), and Sample 2 included 87 infants between 4 and 8 months (32 monolinguals, 25 unimodal bilinguals, and 30 bimodal bilingual infants with a Deaf mother). Eye-tracking was used to analyze infants’ visual scanning of complex arrays including a face and four other stimulus categories. Infants from 4 to 10 months (all groups combined) directed their attention to faces faster than to non-face stimuli (i.e., attention capture), directed more fixations to, and looked longer at faces than non-face stimuli (i.e., attention maintenance). Unimodal bilinguals demonstrated increased attention capture and attention maintenance by faces compared to monolinguals. Contrary to predictions, bimodal bilinguals did not differ from monolinguals in attention capture and maintenance by face stimuli. These results are discussed in relation to the language experience of each group and the close association between face processing and language development in social communication.

## Introduction

From the first days of life, infants attend preferentially to faces and face-like stimuli (Johnson et al., 1991; Valenza et al., 1996; Farroni et al., 2005). These early biases in attention to faces are likely to maximize face experience and social interactions from the very beginning of postnatal life, allowing infants to rapidly develop complex face processing skills.

In older infants, faces continue to capture and maintain attention more than other visual stimuli. Indeed, it has been observed that 6-month-olds direct their first saccade to faces more often than predicted by chance in a complex array comprising a face and multiple visual objects. Increased attention capture by faces compared to objects was also observed in the same experimental design in 7- and 14-month-olds (Elsabbagh et al., 2013). However, increased attention capture by face stimuli was not observed in a similar, but black and white, experimental design in 3- and 6-month-olds (Di Giorgio et al., 2012) or in a color presentation of a face and a toy in 4-to-8-month-olds (DeNicola et al., 2013). Faces are also scanned more extensively than other visual stimuli, attracting a larger number of fixations and increased looking time in 6-month-old infants (Gliga et al., 2009; Di Giorgio et al., 2012) and in 4-to-8-month-olds (DeNicola et al., 2013), but not in 3-month-old infants (Di Giorgio et al., 2012). An increase in attention to faces between 3 and 9 months was observed in a more natural setting where infants watched a cartoon animation (Frank et al., 2009). Interestingly faces had a greater tendency to capture and sustain attention in infants at-risk for autism than infants at low-risk for autism, irrespective of whether these infants were later diagnosed with autism or not (Elsabbagh et al., 2013). Moreover, longer looking time at face stimuli at 7 months was associated with poorer performance in face recognition in 3-year-old infants at-risk for autism (de Klerk et al., 2014). These results are contrary to the idea that autism evolves from an initial lack of attention or interest in social stimuli early in life, but rather suggest complex interactions between social and attentional mechanisms in early development. The level of attention to faces reflects the infant’s interest and processing needs, and higher attention may sometimes associate with processing difficulties.

Although face processing and language acquisition have been traditionally studied in parallel, a few previous studies have suggested that early bilingualism may impact face processing mechanisms in infancy. Different face scanning patterns have been observed for monolingual and bilingual infants when presented with talking faces (Lewkowicz and Hansen-Tift, 2012; Pons et al., 2015). At 4 months, bilinguals show increased attention to the mouth compared to monolinguals. While monolinguals show a preference for looking at the eyes of a talking face, bilinguals show no preference for the mouth or eyes at that age. A strong preference for looking at the mouth of talking faces later develops in monolinguals and bilinguals, and can be observed in both groups at 8 months. At 12 months, monolinguals show preferential looking to the mouth for faces talking in a non-native language, while no preference for the eyes or mouth is observed for the native language. In contrast, 12-month-old bilinguals show a preference for the mouth of faces talking in both native and non-native languages. Moreover, bilingual 8-month-olds are better than monolingual infants of the same age at distinguishing two different languages when silently articulated (Weikum et al., 2007; Sebastián-Gallés et al., 2012), and bilingual infants from 4 to 8 months tend to spend more time looking at talking faces than monolinguals (Mercure et al., 2018). Increased attention to the mouth was also observed for faces displaying non-linguistic emotional movements in 8-month-old bilinguals compared to monolinguals (Ayneto and Sebastian-Galles, 2017), suggesting that bilingualism influences face scanning patterns beyond the context of speech processing. In adulthood, early bilinguals may not demonstrate the classic “other race effect” (Kandel et al., 2016) that is robustly observed in monolinguals (Meissner and Brigham, 2001). These results suggest an impact of early bilingualism on face scanning and face processing.

Unimodal bilinguals acquire two or more spoken languages simultaneously. In other words, these infants acquire two linguistic codes (two sets of sounds, two lexicons, two sets of grammatical rules) and learn to keep them apart, while experiencing a reduced exposure to each of these codes compared to monolinguals (Werker, 2012; Costa and Sebastián-Gallés, 2014). Even though this process is extremely complex, bilingual infants usually reach the milestones of early language development at the same age as monolinguals, including canonical babbling, first word production, and first word combinations (Werker, 2012; Costa and Sebastián-Gallés, 2014). This complex process appears to be made possible by some adaptations in speech and language processing including an increased sensitivity to visual articulation (Sebastián-Gallés et al., 2012) and an increased visual attention to the mouth of talking faces (Pons et al., 2015). Bilingual infants may develop a strategy of orienting to faces faster than monolinguals and scanning them more extensively than monolinguals, which would allow them to make optimal use of articulation cues potentially displayed by these faces. This strategy appears to generalize to contexts in which no speech is present, such as for faces displaying non-linguistic emotional movements (Ayneto and Sebastian-Galles, 2017). This study tests the hypothesis that, compared to monolingual infants, bilingual infants exposed to two spoken languages from birth will demonstrate increased attention capture and attention maintenance for faces in the absence of speech or movement. Attention to faces has never been studied for static faces in bilingual infants. This would suggest that early language experience can impact on attention allocation mechanisms for social stimuli, even in the absence of speech and movement.

A second group of interest in the current study were hearing infants with Deaf mothers. These infants are likely to differ in attention to faces as a result of differences in language and communicative experience. If a Deaf mother uses a sign language, such as British Sign Language (BSL) as her preferred mode of communication, her infant is likely to experience two languages in different modalities. These infants are exposed to a signed language processed mainly in the visual modality (e.g., BSL), and a spoken language processed mainly in the auditory modality (e.g., spoken English). For this reason, they are often referred to as “bimodal bilinguals,” as opposed to “unimodal bilinguals” who are exposed to two spoken languages. Bimodal bilinguals achieve the early linguistic milestones in each of their languages at the same time as children learning two spoken languages (Petitto et al., 2001; Hofmann and Chilla, 2015). Like unimodal bilinguals, bimodal bilinguals may achieve this more complex task by increasing their attention to faces. Congruent with this idea, using eye-tracking, we have previously reported that bimodal bilingual infants spend longer looking at talking faces than monolingual infants (Mercure et al., 2018). Moreover, because infants with Deaf mothers often experience visual forms of communication, visual attention is key to their communicative experience with their mother and other Deaf people in their environment. Sign language communication requires visual attention to the signer and attention to the face appears to be crucial. When presented with sign language, 4- and 14-month-old infants with and without experience of sign language share their visual attention between the signer’s face and hands, but generally spend longer looking at the face than the hands area (Palmer et al., 2012). Similarly, adult signers focus the largest proportion of their visual attention to the face, and not the hands, when perceiving sign language communication (Muir and Richardson, 2005; De Filippo and Lansing, 2006; Emmorey et al., 2008). This increased attention to the face during sign language communication is the hypothesized mechanism for an observed enhancement of certain aspects of face processing in Deaf and hearing signers compared to non-signers (Bettger et al., 1997; McCullough and Emmorey, 1997; Emmorey, 2001; Stoll et al., 2017). Due to the crucial importance of visual attention for sign language communication, Deaf mothers have been observed to use various strategies to obtain visual attention from their child, such as moving in their child’s existing focus of attention (Woll and Kyle, 1989). These patterns of interaction with the mother and with other Deaf communication partners may lead to increased visual attention to the mother, and especially her face (Palmer et al., 2012), in infants of Deaf mothers. Whether their particular experience of communication in the visual modality has an impact on their attention to static faces has never been studied before. We hypothesize that, because of the increased complexity of learning two languages and the increased importance of visual attention in their communication with their Deaf mother, bimodal bilingual infants would demonstrate enhanced attention capture and maintenance for static faces compared to monolinguals and possibly greater than unimodal bilinguals.

The deployment of selective attention in adulthood is not only influenced by perceptual properties of the object (e.g., luminance, contrast, movement), but also by strategies, rewards, and the significance that objects have gained through experience (Chelazzi et al., 2013). Since language experience influences the significance of the face cues in social communication, it is also likely to influence attention to faces. The present study addresses this question by comparing three groups of infants with different language experience. The comparison of two groups of bilinguals – unimodal and bimodal bilinguals – allows distinguishing effects that are caused by learning two languages, from those that are linked to bimodal bilinguals’ unique experience of communication in the visual modality. Visual scanning of complex arrays was studied in two independent samples of infants, following an existing experimental protocol (Gliga et al., 2009; Elsabbagh et al., 2013). Sample 1 compared monolinguals and unimodal bilinguals between 7 and 10 months. Sample 2 compared three groups of 4-to-8-month-old infants with different language experience: monolinguals, unimodal bilinguals, and bimodal bilinguals. It was hypothesized that, compared to monolingual infants, unimodal and bimodal bilinguals would show enhanced attention capture and attention maintenance by faces when they are presented within a complex visual array. It was also predicted that bimodal bilinguals may show this effect to a greater degree that unimodal bilinguals.

## Materials and Methods

### Participants

#### Sample 1

A total of 49 hearing infants between 7 and 10 months contributed data. A further seven infants participated in the study but were excluded due to equipment malfunction or failure to calibrate (*n* = 6), or experimenter error (*n* = 1). Infants were from two groups with different language experience: 26 monolingual infants with hearing parents (16 girls, mean age = 8.7 months), 23 unimodal bilingual infants with hearing parents (6 girls, mean age = 8.4 months). Age did not differ significantly between groups [*F*(1) = 2.0; *p* = 0.159; η^2^ = 0.042]. Monolingual infants were only exposed to English. Both parents were hearing and only used one language. Unimodal bilinguals were frequently and regularly exposed to English and one or more additional spoken language(s). The combination of languages varied between infants. Exposure to each language was estimated by using an English adaptation (Byers-Heinlein, 2009) of the language exposure questionnaire designed by Bosch and Sebastián-Gallés (1997). Unimodal bilinguals were exposed to English on average 52% of the time (standard deviation = 24).

#### Sample 2

A total of 88 hearing infants between 4 and 8 months contributed data. A further seven infants participated in the study but were excluded due to equipment malfunction or failure to calibrate (*n* = 3), withdrawal (*n* = 1), or failure to reach looking time criteria (*n* = 3; see section “Data Analyses”). Infants were from three groups with different language experience: 32 monolingual infants with hearing parents (16 girls, mean age = 6.2 months), 25 unimodal bilingual infants with hearing parents (eight girls, mean age = 6.2 months), and 31 bimodal bilingual infants with a Deaf mother (18 girls; mean age = 6.4 months). Age did not differ between groups [*F*(2) = 0.354; *p* = 0.703; η^2^ = 0.008]. Monolingual infants were only exposed to English. Both parents were hearing and only used one language. Unimodal bilinguals were frequently and regularly exposed to English and one or more additional spoken language(s). The combination of languages varied between infants. All infants in this group had a hearing bilingual/multilingual mother. 18 unimodal bilingual infants also had a bilingual/ multilingual father, and seven had a monolingual father. None reported hearing deficits in any immediate family members. Unimodal bilinguals were exposed to English on average 46% of the time (standard deviation = 23; Byers-Heinlein, 2009). Bimodal bilinguals were frequently and regularly exposed to BSL and English. All infants in this group had a Deaf mother using BSL as her preferred mode of communication; 27 bimodal bilinguals also had a second severely/profoundly D/deaf parent, three had a second parent who was hearing or had mild hearing loss, and one had a single Deaf mother. Bimodal bilinguals were exposed to English on average 40% of the time (standard deviation = 21; Byers-Heinlein, 2009). There was no difference in language exposure to English between the two groups of bilinguals (*p* = 0.311).

Infants with hearing parents (Sample 1 and 2) were contacted from the Birkbeck Babylab database of volunteers recruited from advertisements at mum-and-baby groups, parenting websites and publications. Bimodal bilinguals (Sample 2) were recruited through social media and websites specifically aimed at the Deaf community. Most infants were born at term (37–42 weeks gestation), except for five infants born slightly before term (34–36 weeks) (four monolinguals and one unimodal bilingual: for these infants, a corrected age was used). Parents reported no hearing problems (except for one infant’s mother reporting glue ear) or vision problems (except for one infant’s mother reporting a suspected squint), and no serious mental or physical conditions (except for one infant who had undergone heart surgery). Deaf families were geographically spread across the whole of Great Britain, while infants with hearing parents came mostly from London and surrounding areas. Travel expenses were reimbursed, and a baby t-shirt and certificate of participation were offered to families. This study was carried out in accordance with the recommendations of UCL and Birkbeck Research Ethics Committees. All parents gave written informed consent prior to participation, after explanations of the study in English or BSL depending on the parents’ preferred mode of communication by fluent members of the research team. The protocol was approved by the UCL and Birkbeck Research Ethics Committees and conforms to the Declaration of Helsinki.

### Procedure

Infants from Sample 1 were invited to participate in a larger *Bilingual Babies* research protocol, which began with three eye-tracking tasks presented in TobiiStudio (the “attention to faces” task reported here, as well as tasks investigating audiovisual speech perception and eye gaze perception), followed by seven short eye-tracking tasks on a different experimental set up. The whole protocol usually required between 1 and 1.5 h per infant, including resting, napping, and feeding time. Infants from Sample 2 were invited to participate in the larger *Speak and Sign* research protocol, including a functional near infrared spectroscopy task (investigating brain activation in response to infant-directed spoken and sign language), the same three eye-tracking tasks on TobiiStudio described for Sample 1 and behavioral measures (the Mullen Scales of Early Learning and videos of parent–child interaction). The whole protocol usually required between 1.5 and 3 h per infant, including resting, napping, and feeding time. Only data from the “‘attention to faces” task are reported in the present article. The stimuli and procedures for this task were identical for both samples.

During the “attention to faces” task, infants sat on their parent’s lap in a dimly lit room about 60 cm away from a TobiiT120 eye-tracker (17-in diameter, screen refresh rate 60 Hz, ET sampling rate of 60 Hz, spatial accuracy < 1°). Infant gaze position was calibrated with colorful animations using a five-point routine. Each infant’s gaze and behavior was monitored throughout the study via camera and Tobii Studio LiveViewer. The experimenter occasionally shook a rattle behind the screen to attract the infant’s attention.

### Stimuli

**FIGURE 1:**
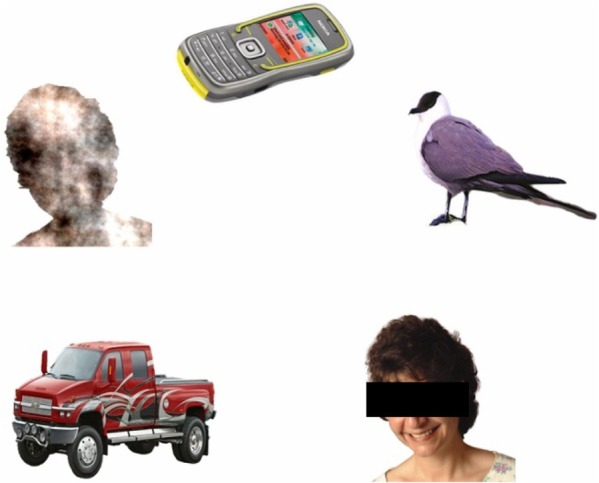
Sample stimulus slides. Face was obscured for publication purposes only.

Five different slides were presented for 10 s each (Gliga et al., 2009; Elsabbagh et al., 2013). In each slide, five color images belonging to five object categories were presented: faces, phase-scrambled faces, birds, cars, and phones (see **Figure 1**). Each individual image was presented only once and the position of each category in the slide was randomized. Images were all of comparable size and presented at an equal distance from the center of the screen. When viewed from a 55 cm distance, the images had an eccentricity of 9.3° and covered an area of approximately 5.2° × 7.3°. Differences in color and luminosity were minimized. Visual saliency (the sensory prominence of an object compared to its background) has been observed to influence visual attention selection mechanisms in adults (Santangelo, 2015), children (Cavallina et al., 2018), and infants (Althaus and Mareschal, 2012). The stimulus categories used in the present study did not differ in terms of visual saliency (Elsabbagh et al., 2013). Faces all had direct gaze and happy expression. There were three female faces and two male faces of different ethnic origins. Scrambled faces were created from each face by randomizing the phase spectra while maintaining the original outer face contour, with the amplitude and color spectra remaining constant. These “attention to faces” slides were interleaved with blocks from other studies.

### Data Analysis

Data were excluded in trials where infants looked at the entire slide for less than 1 s. Only infants with at least three good trials were included in the analyses. These criteria are identical to the ones used by Elsabbagh et al. (2013). Five rectangular regions of interest corresponding to the five categories of objects on each slide were defined in Tobii Studio. Three measures were extracted for each category of objects and averaged for all included trials: fixation latency (the time difference between the beginning of the trial and the beginning of the first fixation to each region of interest), fixation count (the number of fixations within each region of interest), and total fixation duration (the total time spent fixating within each region of interest during the trial period of 10 s). As we did not have any specific hypotheses regarding group differences in attention to birds, cars, phones, and scrambled faces stimuli, the measures for all these stimuli were averaged to create a Non-Face stimulus category. However, any significant Face vs. Non-Face effect was followed by planned comparisons for individual contrasts between Face and each stimulus category to clarify the stability of the effect across control conditions.

## Results

### Fixation Latency

#### Sample 1

**FIGURE 2:**
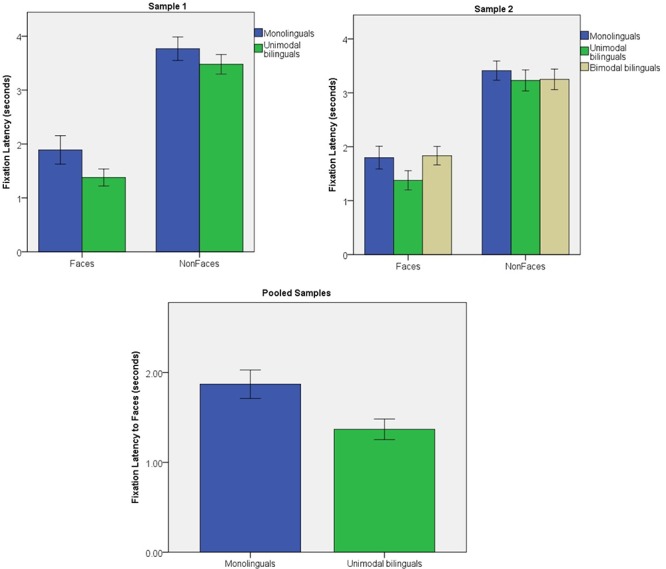
Fixation latency for Face and Non-Face stimuli in Sample 1 and Sample 2; fixation latency for Face stimuli in the pooled samples. Error bars represent standard error.

The latency between the beginning of each trial and the beginning of the first fixation to Face and Non-Face stimuli was analyzed with a stimulus (2) × group (2) ANOVA (see **Figure 2**). A significant effect of stimulus was found [*F*(1,47) = 86.1; *p* < 0.001; η^2^ = 0.647], with Faces attracting infants’ attention faster than other stimulus categories. Individual comparisons of Faces to each stimulus category (birds, cars, phones, and scrambled faces) revealed highly significant effects (all *p* < 0.001). There were no interaction of stimulus × group, but a borderline group effect [*F*(1) = 3.2; *p* = 0.058; η^2^ = 0.075], suggested that unimodal bilinguals tended to orient to both stimulus categories faster than monolinguals. The group effect was significant for Face stimuli [*F*(1) = 4.2; *p* = 0.045; η^2^ = 0.083], but not for Non-Face stimuli [*F*(1) = 0.4; *p* = 0.542; η^2^ = 0.008].

#### Sample 2

Fixation latency was analyzed with a stimulus (2) × group (3) ANOVA (see **Figure 2**). A significant effect of stimulus was found [*F*(1,85) = 124.7; *p* < 0.001; η^2^ = 0.595], with infants orienting faster to Face than to Non-Face stimuli [individual contrasts all *p* < 0.001]. There were no main effect of group [*F*(2) = 1.1; *p* = 0.342; η^2^ = 0.025] or interaction of group × stimulus [*F*(2,85) = 0.6; *p* = 0.572; η^2^ = 0.013]. Group effects were not significant on Face fixation latency [*F*(2) = 1.6; *p* = 0.204; η^2^ = 0.037; *post hoc t*-tests: monolinguals vs. unimodal bilinguals : *p* = 0.391; monolinguals vs. bimodal bilinguals: *p* > 0.999; unimodal vs. bimodal bilinguals: *p* = 0.312].

#### Pooled Analyses

Data from monolinguals and unimodal bilinguals of both studies were pooled together and Face fixation latencies were analyzed in a group (2) × sample (2) ANOVA (see **Figure 2**). Bimodal bilinguals were excluded as they were only present in Sample 2. There was a significant group effect [*F*(1) = 6.2; *p* = 0.014; η^2^ = 0.057]. Overall unimodal bilinguals oriented to faces faster than monolinguals. There were no effect of sample [*F*(1) = 0.1; *p* = 0.741; η^2^ = 0.001], and no interaction of sample × group [*F*(1) = 0.2; *p* = 0.664; η^2^ = 0.002]. The same ANOVA for Non-Face fixations revealed no group effect [*F*(1) = 0.6; *p* = 0.440; η^2^ = 0.006], no sample effect [*F*(1) = 2.1; *p* = 0.145; η^2^ = 0.021] and no interaction of group × sample [*F*(1) < 0.1; *p* = 0.964; η^2^ < 0.001].

### Fixation Count

#### Sample 1

**FIGURE 3:**
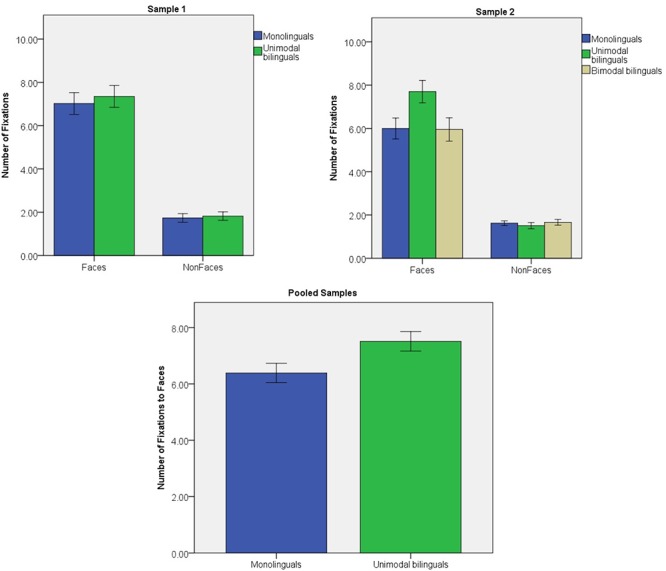
Number of fixations to Face and Non-Face stimuli in Sample 1 and Sample 2; number of fixations to Face stimuli in the pooled samples. Error bars represent standard error.

The number of fixations that infants directed to Faces and Non-Faces was analyzed in a stimulus (2) × group (2) ANOVA (see **Figure 3**). A significant effect of stimulus was found [*F*(1,47) = 188.2; *p* < 0.001; η^2^ = 0.800]. Faces attracted more fixations than any of the other object categories (all *p* < 0.001). There were no main effect of group [*F*(1) = 0.6; *p* = 0.443; η^2^ = 0.013] or stimulus × group interaction [*F*(1,47) = 0.2; *p* = 0.634; η^2^ = 0.005]. A main effect of group was not significant when only Face stimuli were considered [*F*(1) = 0.4; *p* = 0.511; η^2^ = 0.009].

#### Sample 2

The number of fixations was analyzed in a stimulus (2) × group (3) ANOVA (see **Figure 3**). There was a significant effect of stimulus [*F*(1,85) = 235.9; *p* < 0.001; η^2^ = 0.735] with faces attracting more fixations than any of the other objects (all individual contrasts *p* < 0.001). There was a significant interaction of stimulus x group [*F*(2,85) = 3.4; *p* = 0.037; η^2^ = 0.075], but no significant main effect of group [*F*(2) = 3.0; *p* = 0.055; η^2^ = 0.066]. Unimodal bilinguals tended to direct more fixations to faces than the other groups [group effect on Face fixation: *F*(2) = 3.4; *p* = 0.038; η^2^ = 0.074; *post hoc t*-tests: monolinguals vs. unimodal bilinguals: *p* = 0.075; monolinguals vs. bimodal bilinguals: *p* > 0.999; unimodal vs. bimodal bilinguals: *p* = 0.067]. Groups did not differ in terms of fixation to Non-Face stimuli [*F*(2) = 0.2; *p* = 0.699; η^2^ = 0.008].

#### Pooled Samples

After excluding bimodal bilinguals, the number of Face fixations was analyzed for monolinguals and unimodal bilinguals in a group (2) × sample (2) ANOVA (see **Figure 3**). Unimodal bilinguals directed significantly more fixations to Faces than monolinguals [*F*(1) = 4.7; *p* = 0.032; η^2^ = 0.044]. There were no effect of sample [*F*(1) = 0.2; *p* = 0.636; η^2^ = 0.002] and no interaction of sample × group [*F*(1) = 1.7; *p* = 0.201; η^2^ = 0.016].

### Total Fixation Duration

#### Sample 1

**FIGURE 4:**
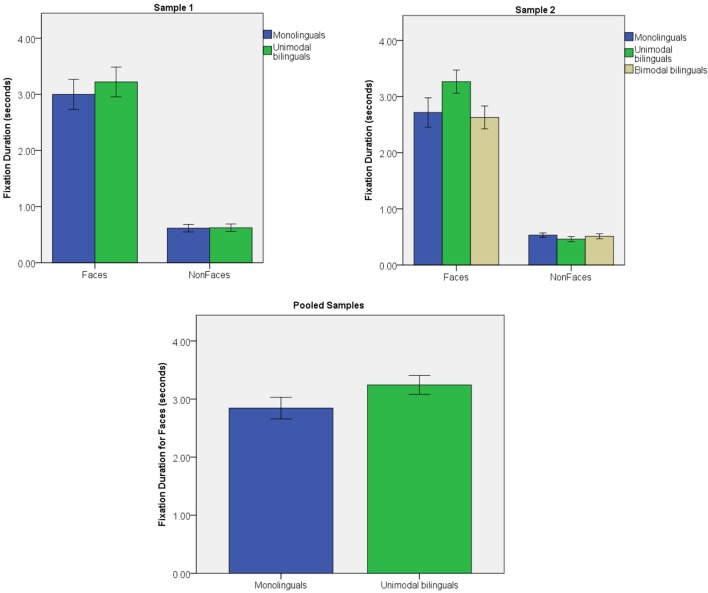
Fixation duration for Face and Non-Face stimuli in Sample 1 and Sample 2; fixation duration for Face stimuli in the pooled samples. Error bars represent standard error.

The total amount of time fixating Faces and Non-Faces over the whole trial was analyzed in a stimulus (2) × group (2) ANOVA (see **Figure 4**). A significant effect of stimulus was found [*F*(1,47) = 135.6; *p* < 0.001; η^2^ = 0.743]. Infants looked at faces for longer than any of the other object categories (all *p* < 0.001). There were no main effect of group [*F*(1) = 0.4; *p* = 0.513; η^2^ = 0.009] or stimulus × group interaction [*F*(1, 47) = 0.2; *p* = 0.622; η^2^ = 0.005]. The main effect of group was not significant when only Face stimuli were considered [*F*(1) = 0.3; *p* = 0.562; η^2^ = 0.007].

#### Sample 2

Total fixation duration was analyzed in a stimulus (2) × group (3) ANOVA (see **Figure 4**). There was a significant effect of stimulus [*F*(1,85) = 283.2; *p* < 0.001; η^2^ = 0.769] with faces being fixated for longer than any of the other objects (all individual contrasts *p* < 0.001). The main effect of group [*F*(2) = 1.8; *p* = 0.175; η^2^ = 0.040], and the stimulus × group interaction [*F*(2,85) = 2.2; *p* = 0.113; η^2^ = 0.050] was not significant. Group effects were not significant on Face fixation duration [*F*(2) = 2.1; *p* = 0.131; η^2^ = 0.047; *post hoc t*-tests: monolinguals vs. unimodal bilinguals: *p* = 0.304; monolinguals vs. bimodal bilinguals: *p* > 0.999; unimodal vs. bimodal bilinguals: *p* = 0.178].

#### Pooled Samples

After excluding bimodal bilinguals, total fixation duration to Face stimuli were analyzed for monolinguals and unimodal bilinguals in a group (2) × sample (2) ANOVA (see **Figure 4**). Unimodal bilinguals tended to spend longer looking at faces than monolinguals, but this difference was not significant [*F*(1) = 3.9; *p* = 0.136; η^2^ = 0.022]. There were no effect of sample [*F*(1) = 0.2; *p* = 0.647; η^2^ = 0.002] and no interaction of sample × group [*F*(1) = 0.4; *p* = 0.522; η^2^ = 0.004].

## Discussion

The present study assessed the influence of early language experience on the development of attention to faces in infancy. Previous literature suggests that faces capture and/or maintain infants’ visual attention more than other stimuli (Gliga et al., 2009; Di Giorgio et al., 2012; DeNicola et al., 2013; Elsabbagh et al., 2013). The present findings are consistent with this literature. Indeed, it was observed that infants from 4 to 10 months orient to faces in a complex visual array faster than they orient to objects or abstract patterns. Infants also directed more fixations at faces than other visual stimuli. They also fixated faces for longer than other visual stimuli.

It was predicted that unimodal bilingual infants would demonstrate increased attention capture and maintenance by face stimuli compared to monolinguals. Consistent with this hypothesis, it was observed that unimodal bilinguals between 4 and 10 months were generally faster at orienting to faces compared to monolinguals. This effect was significant in the pooled samples, and appeared to be more reliable in older infants as it approached significance in Sample 1 (7-to-10-month-olds), but not in Sample 2 (4-to-8-month-olds). Unimodal bilinguals also directed more fixations to faces than monolingual infants of the same age. These effects appeared to be more reliable in younger infants, as it was significant in the younger sample, but not the older sample alone. However, no reliable group differences could be observed in the amount of time infants spent fixating faces. Taken together, these results suggest that unimodal bilinguals direct their attention to faces faster than monolinguals (especially older infants) and that they scan faces more extensively than monolinguals (especially younger infants).

The second hypothesis was that bimodal bilingual infants with Deaf mothers would demonstrate increased attention capture and maintenance by face stimuli compared to monolinguals and potentially unimodal bilinguals. Like unimodal bilinguals, bimodal bilinguals learn two languages, but unlike unimodal bilinguals, they also have a unique experience of communication in the visual modality with their Deaf mother and potentially other Deaf communication partners. Visual attention is key to communication between a Deaf mother and her infant and it has been observed that Deaf mothers deploy strategies to obtain visual attention from their infants (Woll and Kyle, 1989). It was hypothesized that these strategies would act to maximize attention to faces in bimodal bilingual infants, and predicted that increased attention capture and maintenance by face stimuli would be apparent even when presented with static faces. Contrary to hypothesis, bimodal bilinguals did not differ from monolinguals in terms of attention to faces. Bimodal bilinguals oriented to faces faster than to objects and they scanned faces more extensively than objects. However, the magnitude of these effects did not differ from those of monolinguals. It was previously observed that bimodal bilinguals demonstrated increased looking time to talking faces in comparison to monolinguals (Mercure et al., 2018). However, the present results suggest that this effect does not translate to static faces within a complex array. Unlike unimodal bilinguals, bimodal bilinguals do not have to differentiate two spoken languages or learn two systems of speech sounds. The languages that they learn use different sensory modalities and are therefore more easily discriminable. When presented with an unfamiliar static face, bimodal bilinguals do not know whether this is the face of someone using spoken or sign language. The face, but also the hands can be used as cues to discriminate between these languages. For this reason, the presence of a static face without spoken or sign language production may not lead to increased attention to faces in bimodal bilinguals as it does for unimodal bilinguals. However, if the face begins to produce speech, increased attention to the face would be observed in bimodal bilinguals as a strategy to process a language modality in which the infant has less experience than monolinguals (Mercure et al., 2018).

It is important to note that the first hypothesis was tested on data pooled from two independent samples of infants that were collected at two different time points, with a total of 58 monolingual and 48 unimodal bilingual infants. In contrast, the second hypothesis was tested on a single sample of infants collected at one time point, including 32 monolingual and 31 bimodal bilingual infants. Differences in visual attention to faces between monolinguals and unimodal bilinguals were more reliable in the pooled samples than in either of the individual samples. This suggests that there was individual variability in these measures and that analyses benefited from increased sample sizes. Nevertheless, for each of the measures that showed significant group differences in the pooled samples (fixation duration and fixation count), a significant or borderline effect was also observed on one of the individual samples, with a smaller sample size. Due to difficulty in recruiting bimodal bilinguals, it was not possible to recruit a second sample from this special population. However, inspection of **Figures 2**–**4** suggests that attention capture and attention maintenance was highly similar in monolingual and bimodal bilingual infants. Moreover, the *p*-values of the pairwise contrasts between monolingual and bimodal bilingual infants on each of the three measures taken on Face stimuli were larger than 0.999 (see **Table 1**). It is therefore unlikely that significant group differences between monolinguals and bimodal bilinguals would be present in a larger sample size.

**Table 1:** *p*-value of the group differences for Face stimuli for each measure and each experimental sample.

	Monolinguals vs. unimodal bilinguals	Monolinguals vs. bimodal bilinguals	Unimodal vs. bimodal bilinguals
Fixation latency	Sample 1: *p* = 0.045^∗^	*p* > 0.999	*p* = 0.312
	Sample 2: *p* = 0.391 Pooled		
	samples: *p* = 0.014^∗^		
Fixation count	Sample 1: *p* = 0.511 Sample 2:	*p* > 0.999	*p* = 0.067
	*p* = 0.075 Pooled samples:		
	*p* = 0.032^∗^		
Fixation duration	Sample 1: *p* = 0.562 Sample 2:	*p* > 0.999	*p* = 0.178
	*p* = 0.304 Pooled samples:		
	*p* = 0.136		

In adulthood, it has been observed that visual search performance is influenced by rewards associated with each target (Kristjánsson et al., 2010), and selective attention is greatly influenced by the significance that objects have gained through experience (Chelazzi et al., 2013). Unimodal bilingual infants learn that visual cues of articulation are useful to distinguish spoken languages. This is reflected in their increased attention to the mouth of talking faces (Pons et al., 2015), and their increased ability at distinguishing languages based on visual articulation (Sebastián-Gallés et al., 2012). The current data suggest that a unimodal bilingual experience in infancy may reinforce an increased allocation of visual attention to faces, and that this effect could generalize to still faces. Increased visual attention to still faces would allow unimodal bilinguals to take advantage of visual cues of articulation to discriminate different spoken languages if the face was to begin producing speech. It was observed in the present study that unimodal bilinguals scanned faces more extensively than monolinguals and this effect was more pronounced in the younger infant sample (4–8 months). In older infants, this strategy might be modified to orient faster to faces and to engage in extensive scanning for moving/talking faces, but not for still faces. This more sophisticated strategy would allow them to take advantage of visual cues of articulation of talking faces, but would free the infant’s attention to explore other stimuli in the case that articulation cues are not available (for example, in still faces). Congruent with this idea, a faster orientation to faces was observed in unimodal bilinguals compared to monolinguals, and was more pronounced in the older infant sample (7–10 months).

This study demonstrates an impact of language experience on the early development of attention to faces in infancy. The increased complexity of learning two *spoken* languages was found to increase attention capture and maintenance for still faces. These visual strategies may be adaptive to maximize the use of potential visual cues of articulation to allow the discrimination of two spoken languages. Bimodal bilingualism and the experience of communication in the visual modality with a Deaf mother do not appear to impact attention to unfamiliar still faces. Increased attention to faces for bimodal bilinguals compared to monolinguals may be restricted to talking faces in this group (Mercure et al., 2018). Our data suggest that there are complex interactions in the development of face processing and language learning in the context of social communication in infancy.

## Author Contributions

The original idea was conceived by EM, with input from MM, MJ, RF, PB, and TG. The task was designed by TG. Sample 1 was recruited and tested by IQ. Sample 2 was recruited and tested by EM, LG, HB-H, and KC. The data were analyzed by EM with advice from TG, MM, PB, RF, and MJ. The manuscript was written by EM with input from RF, PB, TG, MJ, and MM.

## Conflict of Interest Statement

The authors declare that the research was conducted in the absence of any commercial or financial relationships that could be construed as a potential conflict of interest.
